# Identification of potential biomarkers to differentially diagnose solid pseudopapillary tumors and pancreatic malignancies via a gene regulatory network

**DOI:** 10.1186/s12967-015-0718-3

**Published:** 2015-11-14

**Authors:** Pengping Li, Yuebing Hu, Jiao Yi, Jie Li, Jie Yang, Jin Wang

**Affiliations:** State Key Laboratory of Pharmaceutical Biotechnology, Collaborative Innovation Center of Chemistry for Life Sciences, Jiangsu Engineering Research Center for MicroRNA Biology and Biotechnology, NJU Advanced Institute for Life Sciences (NAILS), School of life sciences, Nanjing University, 163 Xianlin Road, Nanjing, 210023 China; Department of Neurosurgery, Jinling Hospital, School of Medicine, Nanjing University, 305 East Zhongshan Road, Nanjing, 210002 China

**Keywords:** Solid pseudopapillary neoplasms, Gene regulatory network, Shortest path, Biomarker

## Abstract

**Background:**

Solid pseudopapillary neoplasms (SPN) are pancreatic tumors with low malignant potential and good prognosis. However, differential diagnosis between SPN and pancreatic malignancies including pancreatic neuroendocrine tumor (PanNET) and ductal adenocarcinoma (PDAC) is difficult. This study tried to identify candidate biomarkers for the distinction between SPN and the two malignant pancreatic tumors by examining the gene regulatory network of SPN.

**Methods:**

The gene regulatory network for SPN was constructed by a co-expression model. Genes that have been reported to be correlated with SPN were used as the clues to hunt more SPN-related genes in the network according to a shortest path approach. By means of the K-nearest neighbor algorithm (KNN) classifier evaluated by the jackknife test, sets of genes to distinguish SPN and malignant pancreatic tumors were determined.

**Results:**

We took a new strategy to identify candidate biomarkers for differentiating SPN from the two malignant pancreatic tumors PanNET and PDAC by analyzing shortest paths among SPN-related genes in the gene regulatory network. 43 new SPN-relevant genes were discovered, among which, we found hsa-miR-194 and hsa-miR-7 along with 7 transcription factors (TFs) such as SOX11, SMAD3 and SOX4 etc. could correctly differentiate SPN from PanNET, while hsa-miR-204 and 4 TFs such as SOX9, TCF7 and PPARD etc. were demonstrated as the potential markers for SPN versus PDAC. 14 genes were demonstrated to serve as the candidate biomarkers for distinguishing SPN from PanNET and PDAC when considering them as malignant pancreatic tumors together.

**Conclusion:**

This study provides new candidate genes related to SPN and the potential biomarkers to differentiate SPN from PanNET and PDAC, which may help to diagnose patients with SPN in clinical setting. Furthermore, candidate biomarkers such as SOX11 and hsa-miR-204 which could cause cell proliferation but inhibit invasion or metastasis may be of importance in understanding the molecular mechanism of pancreatic oncogenesis and could be possible therapeutic targets for malignant pancreatic tumors.

**Electronic supplementary material:**

The online version of this article (doi:10.1186/s12967-015-0718-3) contains supplementary material, which is available to authorized users.

## Background

Solid pseudopapillary neoplasms (SPN) [1] are uncommon tumors that account for 0.2–2.7 % of all pancreatic tumors and are predominantly seen in young female patients for as-yet-unknown reasons [2]. The fact that SPNs occur predominantly in young women led to the study of gender hormonal receptors by several authors without any evidence of estrogen receptors in pathogenesis of the tumor [3]. Most patients are asymptomatic at diagnosis, and abdominal pain is the most common symptom [3]. SPN shows low-grade malignancy and local surgical excision is usually with a cure rate of greater than 95 % [4, 5]. It is important to distinguish SPN from pancreatic neuroendocrine tumor (PanNET) or pancreatic ductal adenocarcinoma (PDAC) so as to treat them differently, because the treatment of PanNET is usually a selection or combination of surgery, hormone therapy, radiation therapy, and chemotherapy. Similarly, less than 20 % of PDAC patients are suitable for surgery, the gemcitabine or gemcitabine in combination with other chemotherapy agents is thus the main therapeutic measure for PDAC patients [6]. Preoperative diagnosis of SPN will minimize the extent of unnecessary treatment compared with that required for more malignant pancreatic lesions [7, 8]. However, correct diagnosis of SPN is a big challenge because many of the SPN features resembles other types of pancreatic malignant tumors. For example, SPNs are most commonly confused with PanNETs which could occur at pancreatic tail, body and head like SPNs. The difficulty in diagnosis lies in that the two kinds of tumors have histological commonalities, including small- to medium-sized uniform cells with scanty cytoplasm, indiscernible nucleoli, hyaline globules, and numerous small blood vessels with hyalinized walls [9] and both of them behave monomorphous growth and rosette-like structures in morphology [7, 10]. Moreover, both of them can express some neuroendocrine markers, such as CDH1, MME, VIM and CD56 [11]. PDACs which mainly occur at pancreatic head (67 %) with the remaining 33.3 % occur in the body is another type of pancreatic malignancy that has similar radiological features [7, 12] and immunophenotypes to SPN [13].

Recent efforts are devoted to distinguish SPN from other pancreatic tumors at the molecular level and several SPN-related transcription factors (TFs) and other protein-coding genes were exposed. For example, the accumulation of CTNNB1 in nuclear and loss of CDH1 were found to be the characteristic features of SPN. So, immunohistochemical staining of the two proteins could be useful for differentiating SPN from PanNET and PDAC [14, 15]. However, the aberrant behaviors of CTNNB1 and CDH1 in SPN were also observed in some PanNET cases [15, 16]. Similarly, nuclear staining of CTNNB1 and reducing staining of CDH1 could also be positive in some patients with PDAC [13, 17]. Although nearly 30 genes were reported to be SPN related, there have been no genes serving as the gold standard to effectively distinct SPN from malignant tumors in clinical setting.

MicroRNAs (miRNAs) were discovered as a new type of potential biomarker as well as therapeutic targets for diseases in recent years [18]. For example, miR-10b was proposed to be a good diagnostic biomarker for PDAC for its overexpression in the cancer cells [19]. Similarly, upregulation of miR-21 and downregulation of both miR-148a and miR-375 were observed in PDAC relative to adjacent normal tissue and the study therefore proposed that these miRNAs may be used as biomarkers for detecting pancreatic cancer [20]. These small miRNAs encoded by the genome are of about 21nt in length and negatively regulate gene expression by binding to the 3′ UTR of target mRNA and are involved in diverse biological processes, such as differentiation, proliferation, apoptosis etc. [21]. A couple of cancers including colon cancer [22], aggressive B cell lymphoma [23], gastric cancer and invasive endometrial cancer [24] were reported to be associated with specific miRNAs. Park et al. [25] integrated the expression profiles of mRNAs and miRNAs to study the pathogenesis of SPN for the first time. Both mRNAs and miRNAs were shown to be differentially expressed between SPN and PanNET/PDAC. In addition, they found SPN were characterized by three activated pathways, the Wnt/β-catenin, Hedgehog and androgen receptor signaling pathway, with which 17 differently expressed miRNAs were identified to be closely associated by target prediction. However, this work was mainly concerned with the correlation between miRNAs and the pathogenesis of SPNs. It did not elaborate whether miRNAs could be used as biomarkers when discriminating SPNs and the two other pancreatic tumors mentioned above.

Gene regulation is a biological process intertwined by TFs, miRNAs and their target genes, and is vital in controlling gene expression. Abnormal state for certain regulators may affect subsequent regulatory events and thus lead to aberrant cell behaviors. For such a complex system, network has been successfully used as a universal framework to model the biological process in searching genes contributing to the pathogenesis of cancers [26, 27].

In this study, we tried to discover high-quality candidate genes (protein-coding genes and miRNAs) that could diagnose SPN from other malignant pancreatic tumors with the help of the gene regulatory network. The gene regulatory network (GRN) defined here contains three kinds of nodes, including TF, non-TF gene and miRNA. TFs regulate the expression of protein coding genes and miRNAs at the transcriptional level. While miRNAs normally act as negative gene regulators by binding to the 3′UTR of target mRNAs through base pairing, which results in the cleavage of target mRNAs or translation inhibition at the posttranscriptional level. We firstly constructed a GRN by the method that we previously developed and applied to hepatocellular carcinoma [28]. Then we collected the candidate genes related to SPN by searching shortest paths between any pair of known SPN-related genes in the GRN based on the idea that genes interacted together conduct the similar functions [29]. The candidate biomarkers that distinguish SPN from malignant pancreatic tumors were filtered independently from the set of candidate genes by applying K-nearest neighbor algorithm (KNN) on the expression profiles of patient samples. Finally, we evaluated the predictions by the jackknife test. 14 genes including TFs and miRNAs were demonstrated to well separate SPN from PanNET and PDAC samples. The expression patterns of other gene sets were shown to be able to distinguish between SPN and PanNET or SPN and PDAC specifically. So these genes could serve as the potential biomarkers for clinical application. Meanwhile, the discovery of these potential biomarkers may also provide clues to understand the molecular bases of pancreatic tumorigenesis and development as these genes characterize the difference between SPN and PanNET or PDAC.

## Methods

### Microarray data

The mRNA and miRNA expression data were from the SPN study of Park et al. [25] that contained 14 SPN, 6 PanNET, 6 PDAC and 5 non-neoplastic pancreatic samples. We retrieved the data from GEO database [30] with the accession number of GSE 43797. For mRNA, the gene expression profile was obtained using an Illumina HumanHT-12 V4.0 expression beadchip (San Diego, CA). The miRNA data was generated by the Agilent-031181 Unrestricted_Human_miRNA_V16.0_Microarray 030840. Both datasets for mRNA and miRNA expression were log2-transformed and quantile-normalized using the Bioconductor package in R.

### Network construction

We applied a two-step integration method [28] to construct the GRN for SPN, which was previously reported for hepatocellular carcinoma network analysis. The overall procedure for constructing the GRN is briefed as follows. Firstly, a candidate network was obtained through the following steps: (1) predicting target genes for TFs and miRNAs using bioinformatics algorithms, i.e. MATCH [31] for TFs and TargetScan [32] for miRNAs; (2) obtaining experimentally-validated regulations for TFs to targets from ChEA [33] and TransmiR 1.2 [34], miRNAs to targets from TarBase 7.0 [35]; (3) integrating all the predicted regulations and experimentally-validated regulations. Since this step produced a lot of noise and was not restricted to the specific tissue, the co-expression model together with the gene expression profile data was then introduced to pick out the regulatory relationships for the corresponding tissue among the candidate network. The Pearson correlation coefficient for each pair of regulation was calculated at this step and the thresholds (cut-off) were determined according to the power law fitness of the degree distribution to result in a scale-free network [36]. That is, the fraction p(k) of nodes having k connections to other nodes decreases exponentially to k as shown in formula 1. In scale-free networks, a minority of nodes dominates most of the connections. The rationality for the selection of threshold is that many studies have shown the biological networks including protein–protein interaction network [37], metabolic network [38] and regulatory network [39] are of hierarchical scale-free nature. So, the network obtained through this filtration is biologically-meaningful.

1 $${\text{p(k)}} = {\text{k}}^{ - \lambda }$$

where λ is a parameter that typically takes the value of 2 < λ < 3.

### Identifying differentially expressed genes

The two integrated statistical methods: (1) Student’s t-test; (2) median-ratio fold change were used to identify differentially expressed genes for SPN, PanNET and PDAC versus non-neoplastic pancreas samples respectively, SPN versus PanNET and SPN versus PDAC samples. Genes with P value of <0.01 (t-test) and a fold change ≥ 2 or ≤ 0.5 were recognized as differentially expressed genes.

### Previously reported SPN genes and shortest path calculation

Based on the previous studies of immunohistochemical staining, we manually collected genes that have been validated to exhibit abnormal behaviors in SPN using the Polysearch [40], a biomedical text mining tool for extracting relationships between human diseases and genes. After identifying the SPN genes, we calculated the shortest paths for each pair of these genes in the GRN using Dijkstra’s algorithm [41], which was developed to construct shortest paths in weighted networks. In our study, the weight of edges was calculated based on Pearson correlation coefficient, α. For convenience, the parameter β = 1−|α| was taken in the study, so that the smaller the β, the stronger the regulation between two genes.

### Prediction algorithm

The KNN [42, 43] is widely used in computational biology and bioinformatics for its apparent efficiency and easy-to-use features [44, 45]. In the KNN classifier, the query sample should be allocated to the subset represented by majority of its K nearest neighbors. In this study, the KNN classifier was adopted to predict the performance of shortest path genes to classify the three kinds of pancreatic tumors mentioned before, and Euclidean distance was used to measure the locality of samples based on gene expression profiles. As mentioned above, the KNN classifier contains a parameter K that could affect the prediction result. In other words, different K values may assign the query sample to a distinct subset. The one-dimensional method proposed by Kuo-Chen Chou [46] was used to solve this problem. In this method, all samples were classified into M subsets where each subset Sm (m = 1, 2, …, M) is composed of the same attribute category and its size (the number of samples) is Nm. Given a query sample S, it is predicted to belong to the subset S_m_ with which its score of the following equation is the highest.

2 $${\text{Y}}\left( {\text{S}} \right) \, = \sum\limits_{k = 1} {\left[ {{\text{m}}\left( {\text{K,S}} \right) , {\text{S}}_{m} } \right]\left( {{\text{m = 1, 2,}} \ldots , {\text{ M}}} \right)}$$

where

$$\left[ {m({\text{K}},{\text{S}}),{\text{S}}_{m} } \right] = \left\{ {\begin{array}{*{20}l} {1,} &\quad {if\;m({\text{K}},{\text{S}}) \in {\text{S}}_{m} } \\ {0,} &\quad {otherwise} \\ \end{array} } \right\}$$

### Performance validation

In statistical predictions, three kinds of cross-validation methods are widely used to exam the effectiveness of the classifier: the independent dataset test, sub-sampling test (such as fivefold or tenfold cross-validation), and the jackknife test (also called leave-one-out cross-validation) [47]. As demonstrated by Chou.et al. [47], the jackknife test is the least arbitrary and therefore is widely used to examine the performance of various predictors [48–50]. The jackknife test was utilized to evaluate the quality of the prediction model in our study. The prediction accuracy is defined by the percentage of the number of correct prediction events for all classes divided by the number of total prediction events, as follows:

3 $${\rm Accuracy} = \frac{{Total \, number \, of \, correct \, prediction \, number \, events}}{{The \, number \, of \, prediction \, events}} \% .$$

## Results

**Fig. 1:**
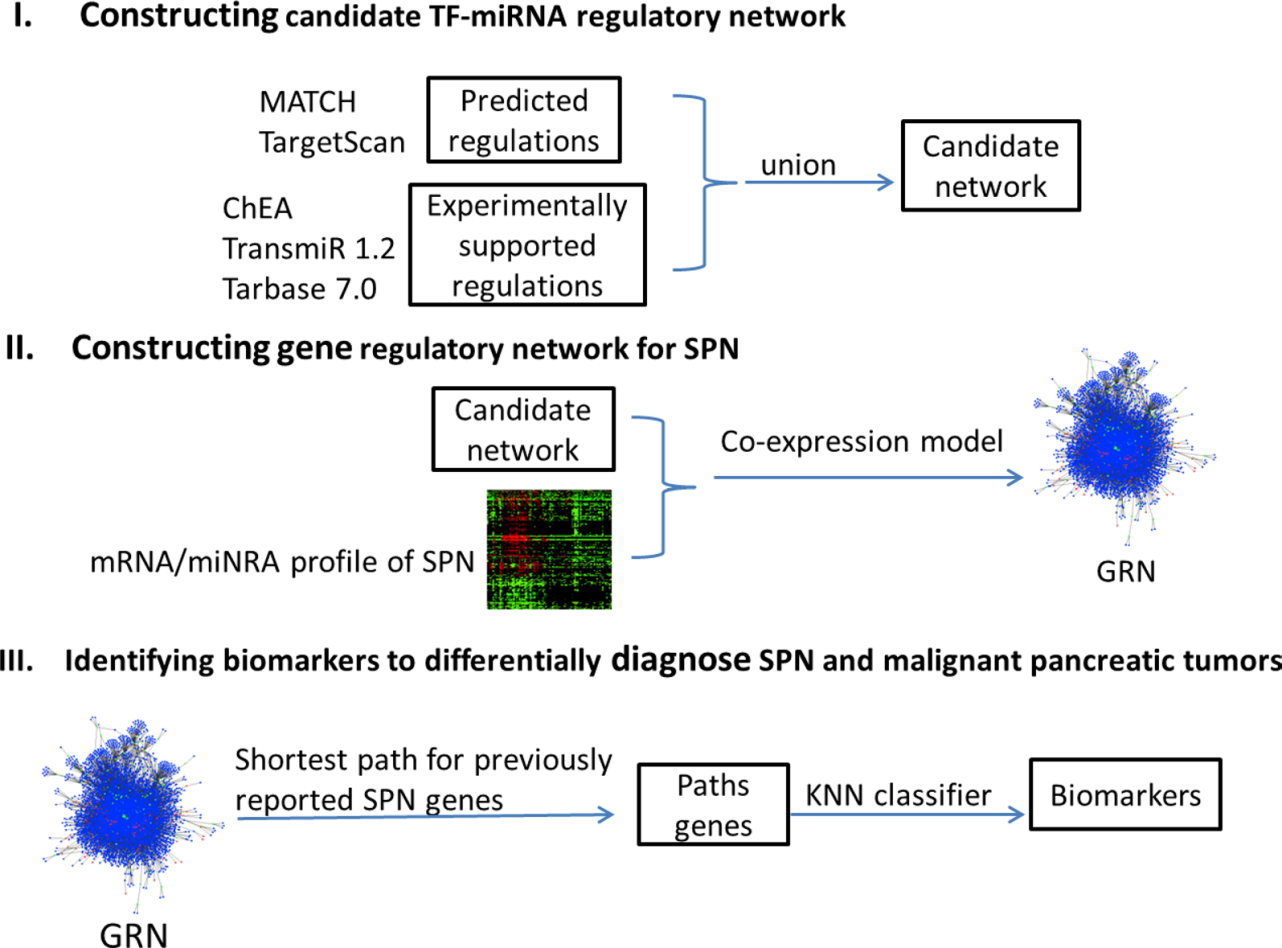
The workflow.** I** The candidate regulatory network was constructed by integrating all the predicted regulations and experimentally-validated regulations conducted by TFs and miRNAs. The bioinformatics tools were listed alongside.** II** To construct the tissue-specific GRN for SPN.** III** Identifying the potential biomarkers by shortest network paths and KNN classifier

To identify high-quality candidate biomarkers that could diagnose SPN from PaNET and PDAC, we firstly constructed the candidate regulatory network by integrated all the predicted regulations and experimentally-validated regulations involving TFs and miRNAs as regulators. Then a tissue-specific GRN for SPN was constructed by integrating co-expression model together with the gene expression profile data. Meanwhile we acquired previously reported SPN gene list (protein coding genes and miRNAs) by Polysearch tool [40] and mapped the members of the list to GRN. By linking the members through shortest path method, we obtained new candidate SPN genes which are on the shortest paths. Finally, the candidate biomarkers that could distinguish SPN from malignant pancreatic tumors were filtered from the set of candidate genes by applying KNN classifier on the expression profiles of patient samples. The workflow is shown in Fig. 1.

### Regulatory network for SPN

**Fig. 2:**
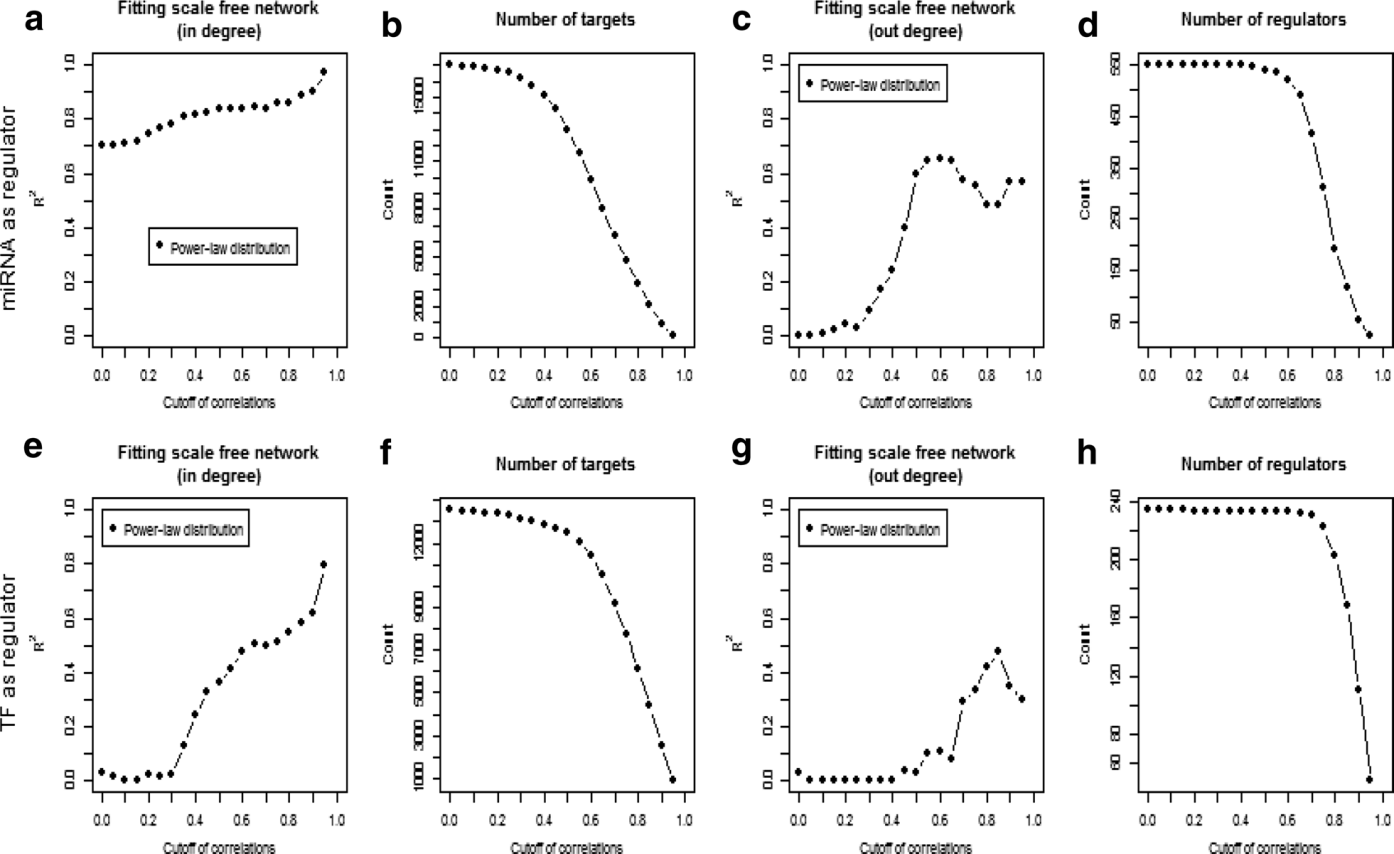
Power-law model fitness to select correlation coefficient thresholds for construction of a GRN in SPN based on the candidate network. The cut-off refers to the absolute value of the correlation coefficient ranging from 0 to 1. The *first row* represents the sub-GRN where only miRNAs are regulators. The *second row* represents sub-GRN where only TFs are regulators. **a**, **e** Plot the results of power law fitting of in-degrees for different cut-off values. The larger R^2^ value, the better power-law fitness. **b**, **f** Number of targets for different cut-off values. **c**, **g** Represent the power-law model of out-degree. **d**, **h** Number of regulators for different cut-off values

To uncover new genes implicated in the pathogenesis of SPN, we constructed the GRN for SPN [28]. The candidate network was firstly constructed by collecting all the regulations of TFs and miRNAs predicted by bioinformatics tools. Then, the co-expression model was applied to this network. In general, the GRN for SPN is constructed by collecting the regulatory interactions between any pair of genes that are co-expressed (see “Methods” section) in SPN. The cut-off parameter used to decide whether there exists a co-expression relationship between specific gene pairs is basically determined by whether the GRN shows a good power-law behavior in degree distribution (Additional file 1). It is shown that the power-law fitness (see R^2^ in Fig. 2a, e) of the in-degree distribution for either miRNA or TF in the GRN of SPN rises as the cut-off for co-expression increases. This means the GRN of SPN approaches the scale-free network when the criterion for co-expression goes strict. However, the strictness of the co-expression criterion also affects the size of the GRN. As seen in Fig. 2b, f, the size of GRN decreases as the co-expression cut-off increases. To avoid losing too many effective regulatory interactions, we set the cut-offs for both TF and miRNA regulation at 0.8 for compromising their impact on the power-law fitness and the size of GRN. It is notable that the SPN analyses based on GRN was basically robust when cut-off varied from 0.6 to 0.8. The final GRN contained 7215 nodes (including 180 TFs and 164 miRNAs) and 86,084 interactions, which comprised 11,351 regulations from miRNAs to protein coding genes, 1013 regulations from TFs to miRNAs and 73,720 regulations from TFs to protein coding genes. Among the GRN, 1182 regulations were experimentally validated. The entire GRN is detailed in Additional file 2.

### Candidate genes that are closely related to SPN

We firstly acquired genes which have been reported to be deregulated in SPN by text mining. Previous studies of SPN were mainly conducted by immunohistochemical staining and have identified various SPN-relevant genes such as FLI1 and CCND1 [51], LEF1 [52] and CTNND1 [53], and CTNNB1 and CDH1 in association with the Wnt signaling pathway [25, 54]. A list of 26 previously reported genes including 4 TFs, 7 miRNAs (Additional file 3: Table S1) were manually extracted by using Polysearch tool [40].

**Fig. 3:**
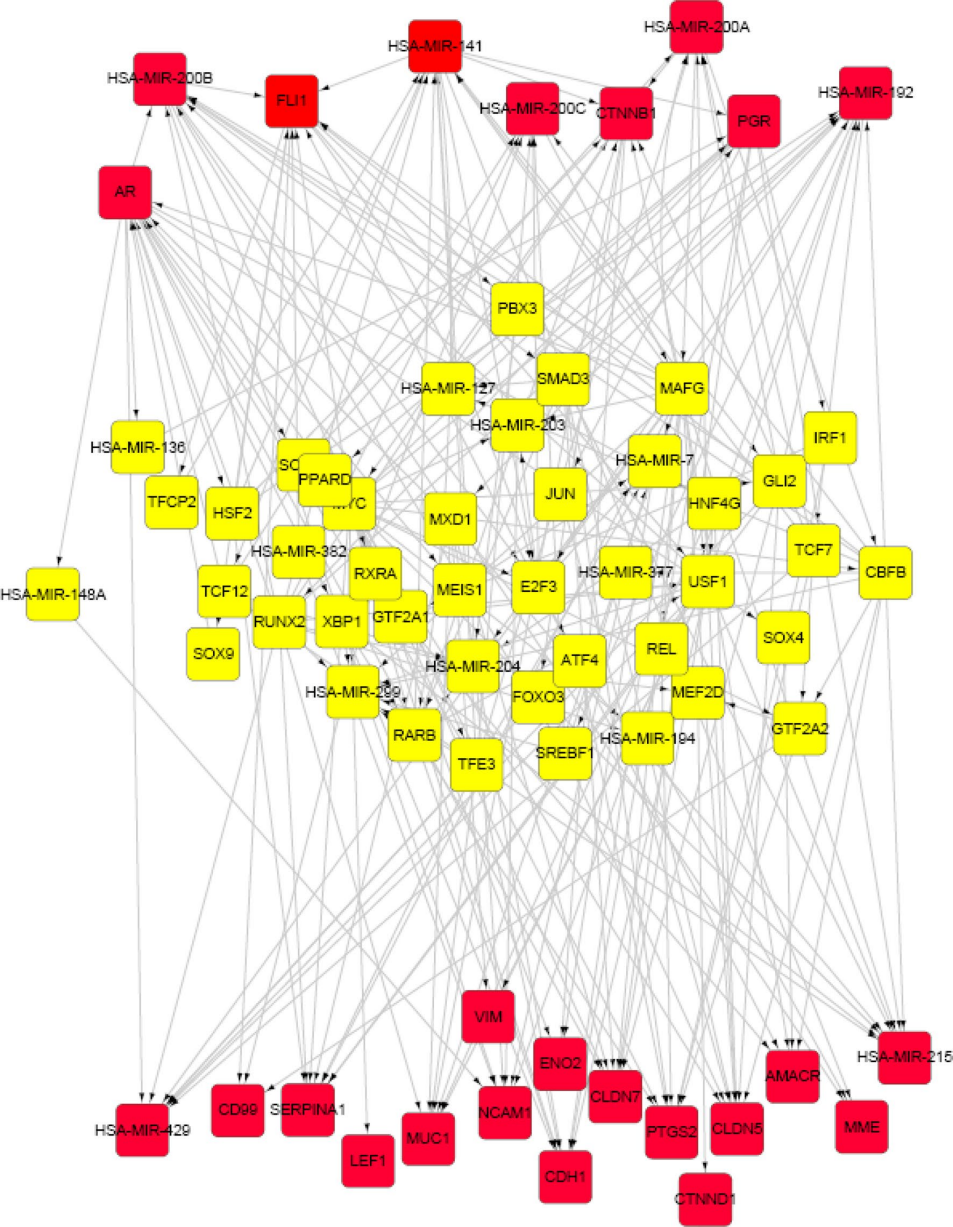
Sub-network constructed by shortest paths. *Red rectangles* represent genes that have already been reported to be related with SPN. *Yellow rectangles* are newly discovered potential SPN-related genes through shortest paths among *red ones*

To discover more candidate genes involved in SPN, we conducted a search in the GRN of SPN based on the “guilt-by-association” rule [29] which has been widely used to predict gene functions in many biological networks [55, 56]. The rule regarded the neighbors of a given gene as to have similar biological functions. According to such a rule, it can be further inferred that genes in the shortest paths [57] between two known SPN genes (i.e. the path with minimal length between two SPN genes) may have features in common with SPN genes. The shortest paths between each pair of the 26 original SPN-related genes were calculated by the algorithm of Dijkstra [41]. A total of 216 shortest paths were obtained (Additional file 3: Table S2), and 43 genes containing 33 TFs and 10 miRNAs were found to be located in the paths (Additional file 3: Table S3) in addition to those 26 known SPN genes. The 216 shortest paths formed a sub-network (Fig. 3, 25 known genes were shown in the figure, as there was no shortest path between CCDN1 and the other known genes) in which, transcription information is transmitted among known SPN-related regulators and 43 path genes. These 43 genes were believed to be new candidates implicated in the tumorigenesis of SPN according to “guilt-by-association” rule.

### Functional analysis for candidate SPN genes

**Fig. 4:**
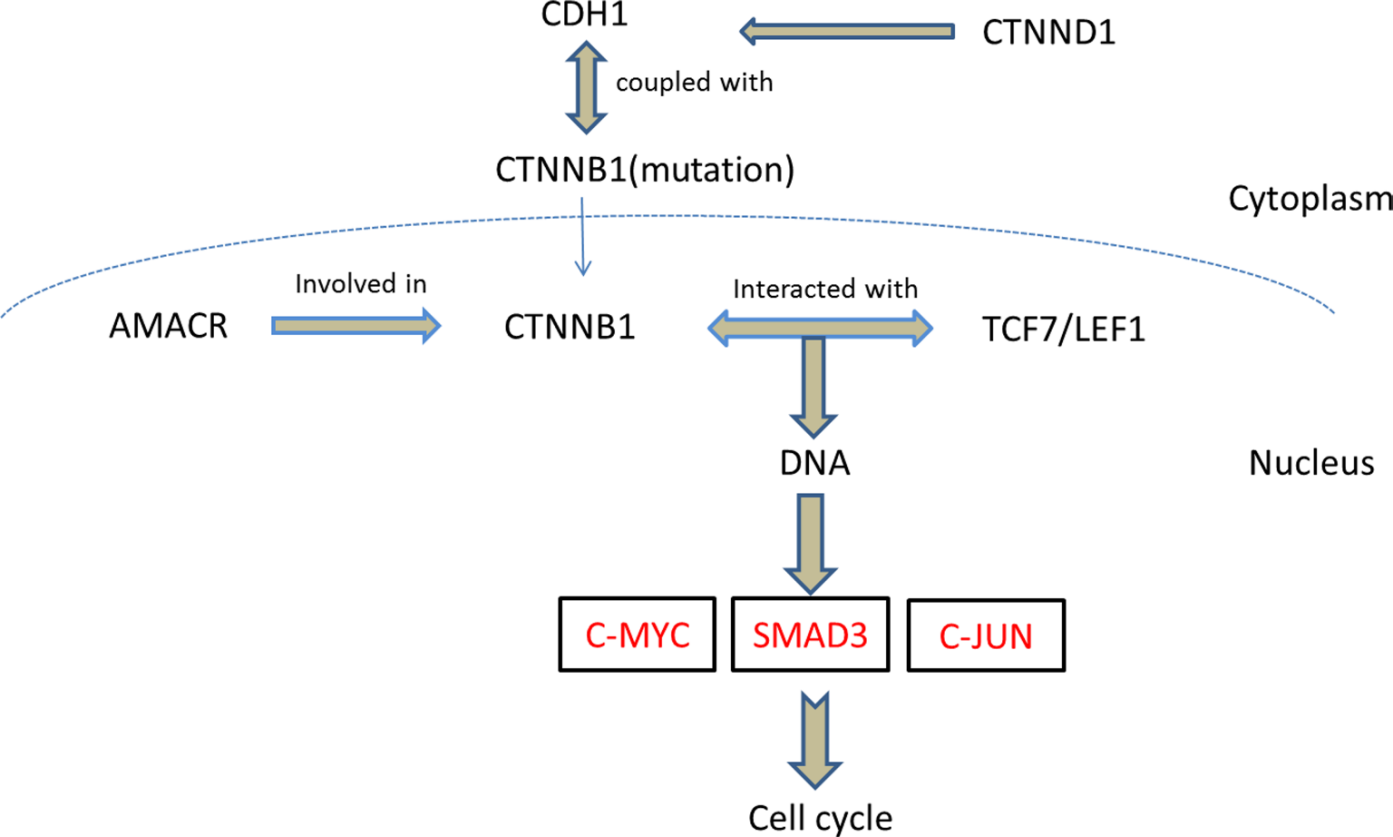
Wnt signaling pathway involved in SPN. Genes in *box* were found to be deregulated in the Wnt signaling pathway

Functional analyses were made to testify whether the new candidate genes were truly correlated with SPN. Firstly, we performed function enrichment analysis on the candidate TFs. KEGG pathway enrichment analysis demonstrated that all of the candidate TFs were involved in classic cancer-related pathways, such as non-small cell lung cancer, and colorectal cancer (Table 1). Specially, some of genes were enriched in the Wnt signaling pathway whose activation has been reported to be the essential characteristics for the pathogenesis of SPN [13, 25, 51]. Figure 4 illustrates the genes deregulated in Wnt signaling pathway, where SMAD3, c-MYC and c-JUN which participated in cell cycle were found aberrantly expressed in SPN when comparing to non-neoplastic pancreas samples.

**Table 1:** KEGG enrichment analysis for 33 new candidate TFs

Term	Genes	p value	Benjamini
hsa05200: Pathways in cancer	E2F3, TCF7, PPARD, JUN, RXRA, SMAD3, RARB, GLI2, MYC	5.88E−07	2.29E−05
hsa05223: Non-small cell lung cancer	E2F3, RXRA, RARB, FOXO3	3.79E−04	0.007373
hsa04310: Wnt signaling pathway	TCF7, PPARD, JUN, SMAD3, MYC	5.93E−04	0.00768
hsa05210: Colorectal cancer	TCF7, JUN, SMAD3, MYC	0.001388	0.013451
hsa05222: Small cell lung cancer	E2F3, RXRA, RARB, MYC	0.001388	0.013451
hsa05216: Thyroid cancer	TCF7, RXRA, MYC	0.002739	0.021169

In addition, the functions of 10 candidate miRNAs were annotated by the tool TAM [58], a web-accessible program which could mine the potential biological processes that a set of miRNAs could be involved in. The results showed miRNA-associated functions were enriched in apoptosis, cell differentiation and epithelial-mesenchymal transition (EMT) (P < 0.05). All of these functions are also closely related to the tumorigenesis.

### Candidate genes were enriched in differentially expressed genes

Basically, the genes that contribute to the pathogenesis and development of the disease are prone to be differentially expressed in SPN comparing to the normal state. We checked the expression value of all the candidate genes in both SPN and normal condition and found that most of the candidate genes (TFs and miRNAs) were differentially-expressed (20 out of 33 TFs, 6 out of 10 miRNAs, Tables 2, 3). Taking the 48/41 TFs/miRNAs (see “Methods” section and Additional file 4) that are differentially expressed in SPN as background, the Fisher’s exact test showed that both the candidate TFs and miRNAs are significantly enriched in differentially-expressed genes (P < 0.05). This suggests that the candidate genes identified by the shortest-path method are generally essential to the pathogenesis of SPN.

**Table 2:** Overlap for differentially-expressed TFs and candidate TFs

	DETs	Non-DETs	Total
Path TFs	20	13	33
Background TFs	48	132	180

Fisher’s exact test (*P* < 0.05)

*DET* differentially-expressed TFs

**Table 3:** Overlap for differentially-expressed miRNAs and candidate miRNAs

	DEMs	Non-DEMs	Total
Path miRNAs	6	4	10
Background miRNAs	41	881	992

Fisher’s exact test (*P* < 0.05)

*DEM* differentially-expressed miRNAs

### Candidate biomarkers to differentially diagnose SPN and malignant pancreatic tumors

**Fig. 5:**
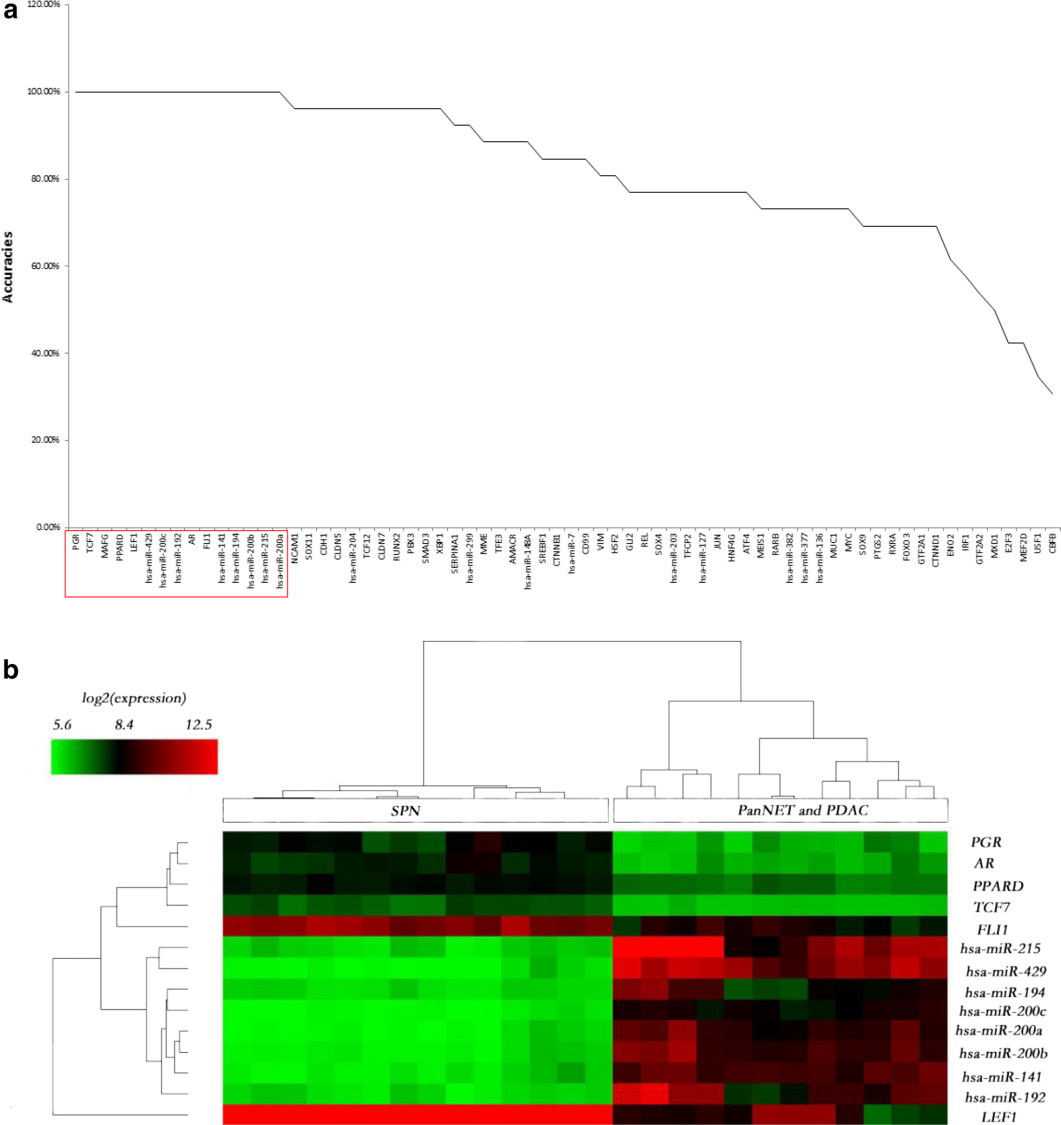
Biomarkers for SPN and malignant cancers. **a** Plot of the KNN classification accuracy for 69 genes independently in differentiating SPN from malignant cancers. **b** Heatmap of expression profiles of potential biomarkers for SPN and malignant cancers. *Rows* correspond to genes and *columns* correspond to samples. *Color bar gradient* represents log2 expression

The candidate biomarkers that could separate SPN from malignant pancreatic tumors (including PanNET and PDAC) were searched in the 69 SPN-related genes, i.e. the 26 known genes collected from literatures and the 43 new candidate genes that were predicted in this study. The procedure is as following: (1) the KNN classifier was applied on gene expression profiles of the 69 SPN-related genes to find out the gene set that could independently distinguish SPN and malignant pancreatic tumors; (2) the quality of the KNN classifier was evaluated by jackknife test (see Method). 14 genes were found to discriminate SPN from pancreatic malignancies with 100 % accuracy (Fig. 5a, b) when taking PanNET and PDAC as a whole, among which 8 genes were downregulated in SPN while 6 other genes were upregulated compared with PanNET as well as PDAC. Three genes, TCF7, PPARD and miR-194, were newly found in this study (Additional file 5: Table S6). Obviously, the expression patterns of the genes found here represent the difference between SPN and the other two malignant cancers and may help to understand the molecular mechanisms between benign neoplasms like SPN and malignant tumors.

**Fig. 6:**
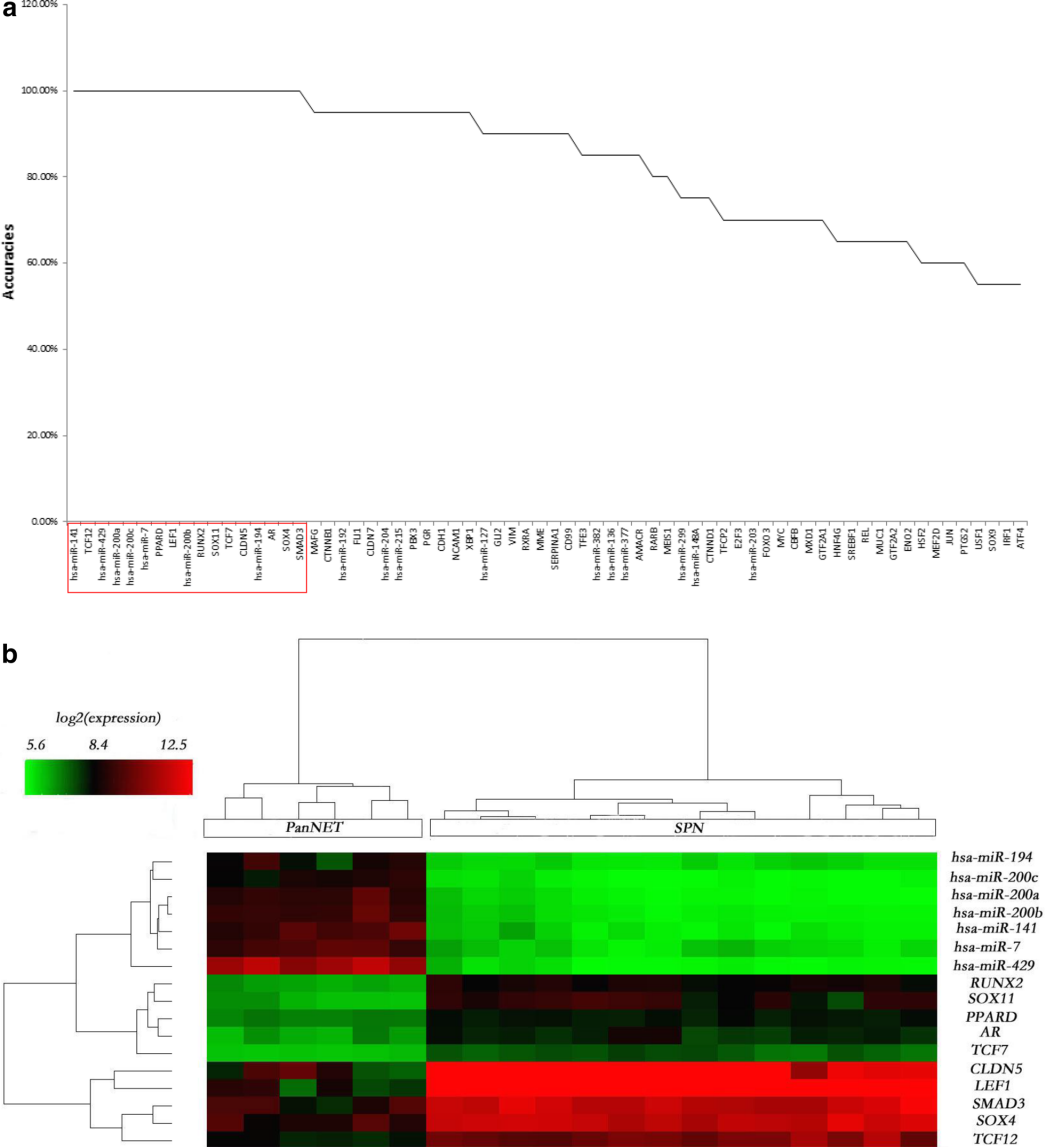
Biomarkers for SPN versus PanNET/PDAC. **a** Plot of the KNN classification accuracy for 69 genes independently in differentiating SPN from PanNET. **b** Heatmap of expression profiles of biomarkers for SPN and PanNET. *Rows* correspond to genes and *columns* correspond to samples, *color bar gradient* represents log2 expression

**Fig. 7:**
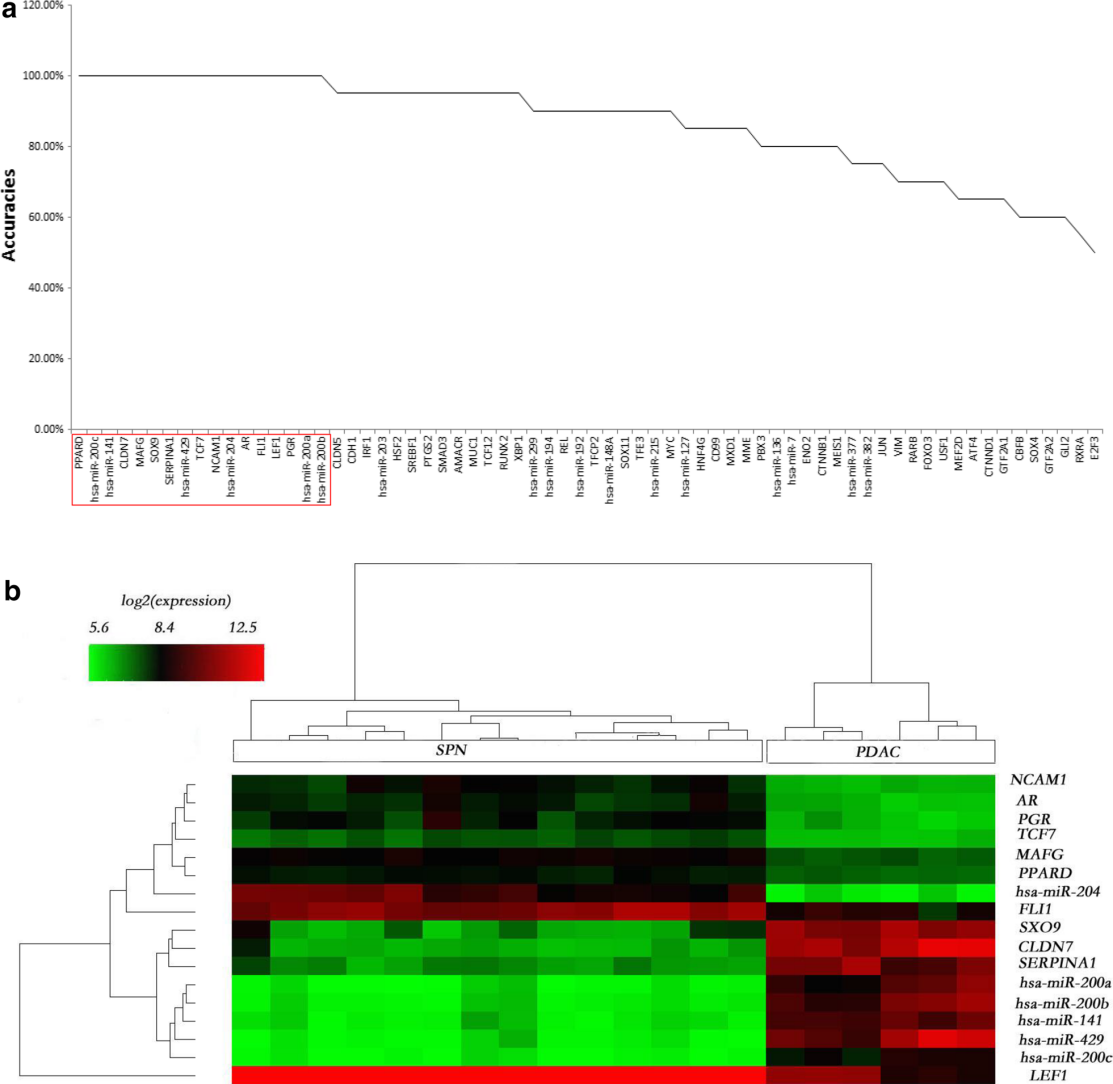
Biomarkers for SPN versus PanNET/PDAC. **a** KNN classification accuracy for 69 genes independently in differentiating SPN from PDAC. **b** Heatmap of expression profiles of potential biomarkers for SPN and PDAC

Particularly, we searched the candidate biomarkers for discriminating SPN from PanNET or PDAC respectively among the 69 SPN-related genes following the same procedure. 17 genes were identified to separate SPN from PanNET with 100 % accuracy (Fig. 6a, b) and 7/10 of them decreased/increased in SPN when comparing with PanNET. The same number of genes and accuracy were obtained for SPN versus PDAC (Fig. 7a, b) with 8/9 genes decreased/increased in SPN compared to PDAC. For the distinction of SPN from PanNET, 9 genes were newly predicted; while for the distinction of SPN from PDAC, 5 genes are newly found (Additional file 5: Table S7/S8). Comparing the two sets of 17 candidate biomarkers, it is found that TCF7 and PPARD are the common members (Figs. 6b, 7b) and both of them were upregulated in SPN compared with PanNET and PDAC (Additional file 5).

## Discussion

In this study, we conducted a first investigation to identify the potential biomarkers for differentially diagnosing SPN from malignant pancreatic tumors, PanNET and PDAC, via a network approach. The shortest paths among 26 previously-reported SPN-related genes in the network were calculated and 43 new candidate genes were identified. Genes from this analysis together with the previously reported SPN-related genes may potentially contribute to the pathogenesis of SPN and help to the precise diagnosis of SPN which is important to improve the prognosis. Thus, we further explored candidate biomarkers for the differentiation of SPN from malignant pancreatic tumors using a nearest neighbor algorithm that was evaluated by the jackknife test.

When considering PanNET and PDAC collectively as malignant pancreatic cancers, *PPARD*, *TCF7* and miR-194 were found to have excellent capabilities to separate SPN from malignant cases. More interestingly, miR-194 was down-regulated in SPN (−2.76-fold, *P* < 0.01), but was up-regulated in both PanNET (2.06-fold, *P* < 0.01) and PDAC (2.97-fold, *P* < 0.01) when all classes were compared with non-neoplastic pancreatic cases. A recent study revealed that up-regulation of miR-194 in PDAC was correlated with increased tumor growth and progression [59], which supports our results.

For SPN versus PanNET, we uncovered a set of 17 genes including 9 new candidate genes and 8 previously-reported genes that could correctly separate the two groups of samples (14 SPN and 6 PanNET). Of the path genes, sex-determining region Y-box 11 (SOX11; 5.62-fold versus normal, 5.02-fold versus PanNET, *P* < 0.01), which has an out-degree of 690 in the network was reported to be implicated in embryonic development and tissue remodeling [60]. In this study, SOX11 was found to serve as a candidate biomarker to confer a differential diagnosis between SPN and PanNET. SOX11 was also found to be exclusively overexpressed in SPN when the gene expression profiles of SPN with non-neoplastic samples, PanNET with non-neoplastic samples, and PDAC with non-neoplastic samples were compared. Interestingly, in epithelia ovarian cancer (EOC), *SOX11* was revealed to be overexpressed when compared with normal ovarian tissues, but loss of expression of SOX11 protein was associated with a more aggressive phenotype [61]. The hypothesis that SOX11 expression in EOC may lead to the aberrant regulation of genes associated with cell survival/death which could promote a pro-apoptotic and less aggressive phenotype was thus postulated in that report. It has also been reported that the overexpression of SOX11 strongly suppresses cell migration/invasion in vitro and in vivo but does not inhibit cell proliferation in gastric cancer [62]. Given these facts, we speculated that overexpression of SOX11 may contribute to the tumorigenesis but less malignant behaviours of SPN, although the detailed mechanisms need further investigation.

Another set of 17 genes consisting of five new candidate genes and 12 reported genes were found to successfully differentiate SPN from PDAC. Of the path genes, sex-determining region Y (SRY) box 9 (SOX9) has an out-degree of 2030 in the network. SOX9 is an important transcription factor required for development and has been implicated in several types of cancer. SOX9 decreased in SPN as compared with PDAC (−12.62-fold, P < 0.01) and non-neoplastic pancreatic tissues (−10.73-fold, P < 0.01), which was consistent with the previous immunohistochemistry data that the detection of SOX9 was observed in the majority (89 %) of PDACs samples but not in SPN (0 %) [63]. Furthermore, SOX9 was also found to be critical for PDAC initiation and involved in their tumorigenesis by regulating the ERBB pathway [64]. These facts implied that SOX9 could act as a candidate biomarker in differentiating SPN and PDAC.

Another path gene that could successfully differentiate SPN from PDAC was miR-204, which increased in SPN compared with PDAC (10.86-fold, *P* < 0.01) and non-neoplastic pancreatic tissues (5.65-fold, *P* < 0.01). MiR-204 has been reported to be down-regulated in several human cancers, including gastric cancers, ovarian cancers, breast cancers, malignant peripheral nerve sheath tumors as well as endometrial cancers, and associated with the promotion of tumor invasion and metastasis [65]. Additionally, overexpression of miR-204 dramatically suppressed intrahepatic cholangiocarcinoma cell migration and invasion, as well as the EMT process [66]. Aberrant EMT activation is an important step towards tumor cell invasion and metastasis [67, 68]. From these discoveries, we inferred that miR-204 may be a vital factor to maintain SPN in a low malignant state, and this mechanism requires further study of course. Since miR-204 was down-regulated in PDAC compared with non-neoplastic pancreatic tissues (−2.01-fold, *P* < 0.01), we speculated whether miR-204 could be of therapeutic usage to suppress tumor metastasis in PDAC. In fact, over-expression of miR-204 was found to cause cell death in malignant pancreatic cancer [69].

The strategy we proposed to discover candidate biomarkers to discriminate SPN from PanNET or PDAC was to search for the candidate SPN-related genes through SPN gene regulatory network firstly, and then to analyze the expression profiles by focusing on the resulted SPN genes in order to decrease the noise that most of the current large scale gene expression analysis are confronted with. The strategy worked well with 100 % accuracy for the dataset available so far. However, it should be noted that more samples should be included to further quantify the importance of each marker gene in the future. Despite the shortage of the amount of samples, the effectiveness of this approach has been demonstrated by showing the biological relevance of the candidate biomarkers as well as the experimental evidence from literatures for certain genes. The potential biomarkers including miRNAs and TFs proposed here need further validation by qRT-PCR, immunohistochemical staining and Western blotting, etc. In fact, mRNA level of one candidate biomarker, androgen receptor (AR) has been verified by qRT-PCR in patient samples [25]. Moreover, both western blotting and immunohistochemical staining analyses revealed that AR was increased in SPN comparing to PanNET and PDAC by Park et al. [25]. Once the potential biomarkers are confirmed, they may be adapted to clinical setting for differential diagnosis between SPN and PanNET/PDAC in which tumor tissue section will be used and the expression level of potential marker genes be checked.

## Conclusions

In conclusion, this study provides new insights into the identification of new potential biomarkers to differentiate SPN from PanNET as well as PDAC by a network-based study. 43 new candidate genes involved in the tumorigenesis of SPN were found by using the shortest path analysis among 26 reported SPN-related genes. With the help of KNN classifier and jackknife test, 14 genes were found to discriminate SPN from pancreatic malignancies with 100 % accuracy, and three genes, TCF7, PPARD and miR-194 were newly found in this study. For SPN versus PanNET, 17 genes including SOX11, SMAD3 and miR-194 etc. were identified to separate the two diseases, among which nine were newly discovered. The same number of genes and accuracy were obtained for the distinction of SPN from PDAC, with five genes containing SOX9 and miR-204 etc. were newly predicted. Genes obtained from this study may provide clues to further understanding of the gene regulation mechanism of SPN as well as PanNET and PDAC. Some potential biomarkers, e.g. SOX11 and miR-204 which could cause cell proliferation but inhibit invasion or metastasis could be potential therapeutic targets for malignant pancreatic tumors to lighten their malignant degree.

## Additional files

10.1186/s12967-015-0718-3 In-degree distribution for GRN. X-axis represents the in-degree for a certain node. A node of in-degree x means that this node is regulated by a total number of x other nodes. Y-axis represents the total number of network nodes which has an in-degree of x. The red curve was the fitting to the power law distribution. (A): The in-degree distribution for sub-GRN in which only miRNAs are included as regulators and the in-degree for each node (miRNAs and protein coding genes) was calculated in this sub-GRN. The in-degree ranges from 0 to 27. (B): In-degree distribution for sub-GRN in which only TFs are included as regulators.
10.1186/s12967-015-0718-3 Gene regulatory network of SPN. The first column represents regulators, while the second column represents corresponding targets.
10.1186/s12967-015-0718-3 Previously reported SPN-relevant genes and shortest paths among path genes. Table S1 lists the 26 genes which have been reported to be related with SPN. Table S2 shows in detail the shortest paths for each pair of 26 SPN-related genes listed in table S1. Table S3 lists 43 new candidate genes on the shortest path, together with the corresponding gene type.
10.1186/s12967-015-0718-3 Differentially expressed genes between SPN and non-neoplastic samples. Table S4 posts differentially expressed TFs with fold change and t-test p-value for each TF. Table S5 posts differentially expressed miRNAs with fold change and t-test p-value too for each miRNA.
10.1186/s12967-015-0718-3 Biomarkers for differential diagnosis of SPN and two malignant pancreatic. Table S6 lists fold change of 14 biomarkers for SPN, PanNET and PDAC versus normal pancreatic tissues as well as SPN versus PanNET and PDAC. Table S7 lists fold change of 17 biomarkers for SPN and PanNET versus normal pancreatic tissues as well as SPN versus PanNET. Table S8 lists fold change of 17 biomarkers for SPN and PDAC versus normal pancreatic tissues as well as SPN versus PDAC.
