# Supplementary figures and images for: Identification of potential biomarkers to differentially diagnose solid pseudopapillary tumors and pancreatic malignancies via a gene regulatory network
Source: J Transl Med. 2015 Nov 14;13:361. doi: 10.1186/s12967-015-0718-3 (PMC4650856; doi:10.1186/s12967-015-0718-3)

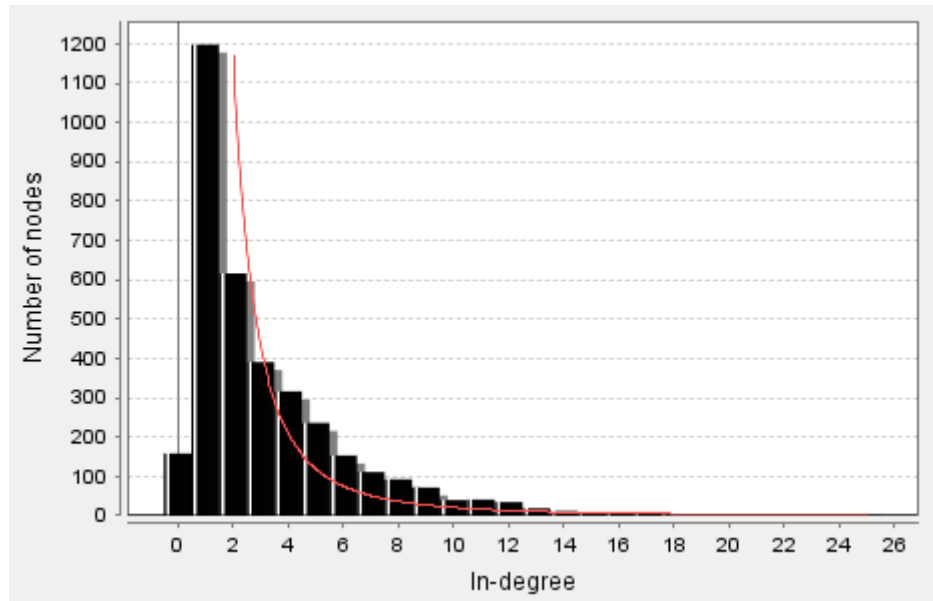

A

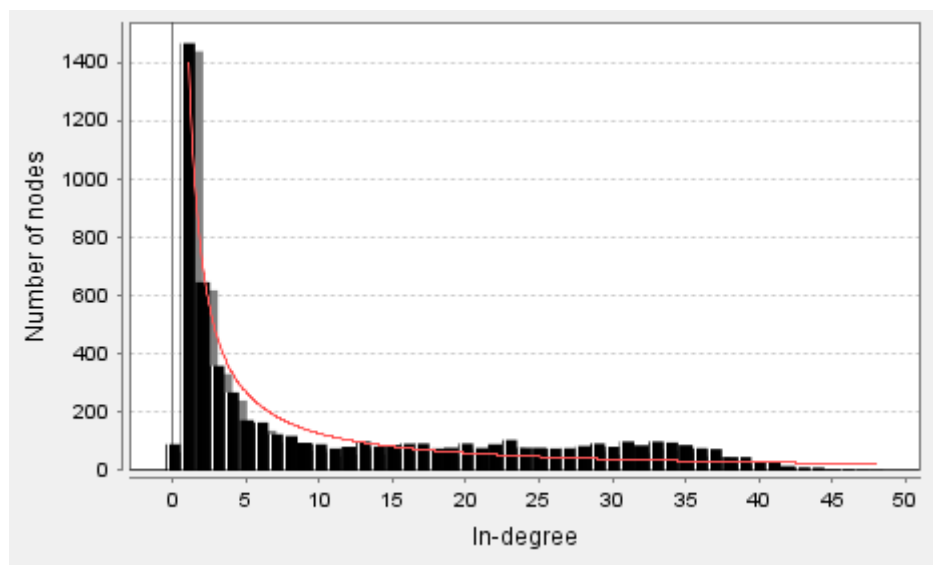

B

Supplement: Supplementary file 1 — 10.1186/s12967-015-0718-3 In-degree distribution for GRN. X-axis represents the in-degree for a certain node. A node of in-degree x means that this node is regulated by a total number of x other nodes. Y-axis represents the total number of network nodes which has an in-degree of x. The red curve was the fitting to the power law distribution. (A): The in-degree distribution for sub-GRN in which only miRNAs are included as regulators and the in-degree for each node (miRNAs and protein coding genes) was calculated in this sub-GRN. The in-degree ranges from 0 to 27. (B): In-degree distribution for sub-GRN in which only TFs are included as regulators. [file 12967_2015_718_MOESM1_ESM.pdf]
